# β-catenin in plants and animals: common players but different pathways

**DOI:** 10.3389/fpls.2014.00143

**Published:** 2014-04-10

**Authors:** Manisha Sharma, Amita Pandey, Girdhar K. Pandey

**Affiliations:** Stress Signal Transduction Lab, Department of Plant Molecular Biology, University of Delhi South CampusNew Delhi, India

**Keywords:** beta-catenin, Armadillo, abiotic stress, Wnt signaling, U-box

## Introduction

A key node in number of essential cellular processes in eukaryotes, Armadillo was originally characterized in Drosophila as the component of Wingless/Wnt signal transduction pathway (Nusslein-Volhard and Wieschaus, 1980). β-catenin is the mammalian homolog of Armadillo playing dual role in structural and transcriptional regulation during embryonic development (Conacci-Sorrell et al., 2002). Even though initially characterized in animals, members of the Armadillo proteins are also known to exist in non-animals including slime mold (*Dictyostelium discoideum*) and plants (Wang et al., 1998; Barelle et al., 2006; Veses et al., 2009). The existence of Armadillo repeat family of proteins across species suggests ancient evolutionary origin and functional conservation of these proteins in multicellular organisms (Coates, 2003). The intricate role of β-catenin raises several doubts about the mechanism by which it mediates interaction with diverse partner proteins using common interface, and how this interaction influences adhesion and transcription?

The ARM family proteins have been identified with multiple functional domains in more than one species. Genome-wide studies in plants have shown the existence of large number of Armadillo homologs in *Physcomitrella patens*, Arabidopsis and *Oryza sativa* (Mudgil et al., 2004; Sharma et al., 2014). One assumption is that, Armadillo family being evolutionary conserved, perform similar role in all organisms. However, the existence of multigene Armadillo family with various subfamilies indicate novel species specific functions of these proteins in plants. Several recent studies have made known the function of numerous ARM proteins in Arabidopsis and rice. Apart from their analogous role in regulation of gene expression and developmental processes, various proteins were discovered to be predominantly involved in plant stress responses.

Thus, an intriguing and important question remains as in what way the similar effector proteins of Wnt pathway function and how similar canonical response is prevented or exist in plants. Recent progress in studies of ARM proteins in plants has suggested some possible answers to this question. However, the Wnt signaling mechanism regulated by ARM repeat proteins is still unknown. Regarding this, many underscoring questions are just beginning to emerge that remains to be answered.

## Wnt signaling—developmental regulation in plants and animals

**Figure 1:**
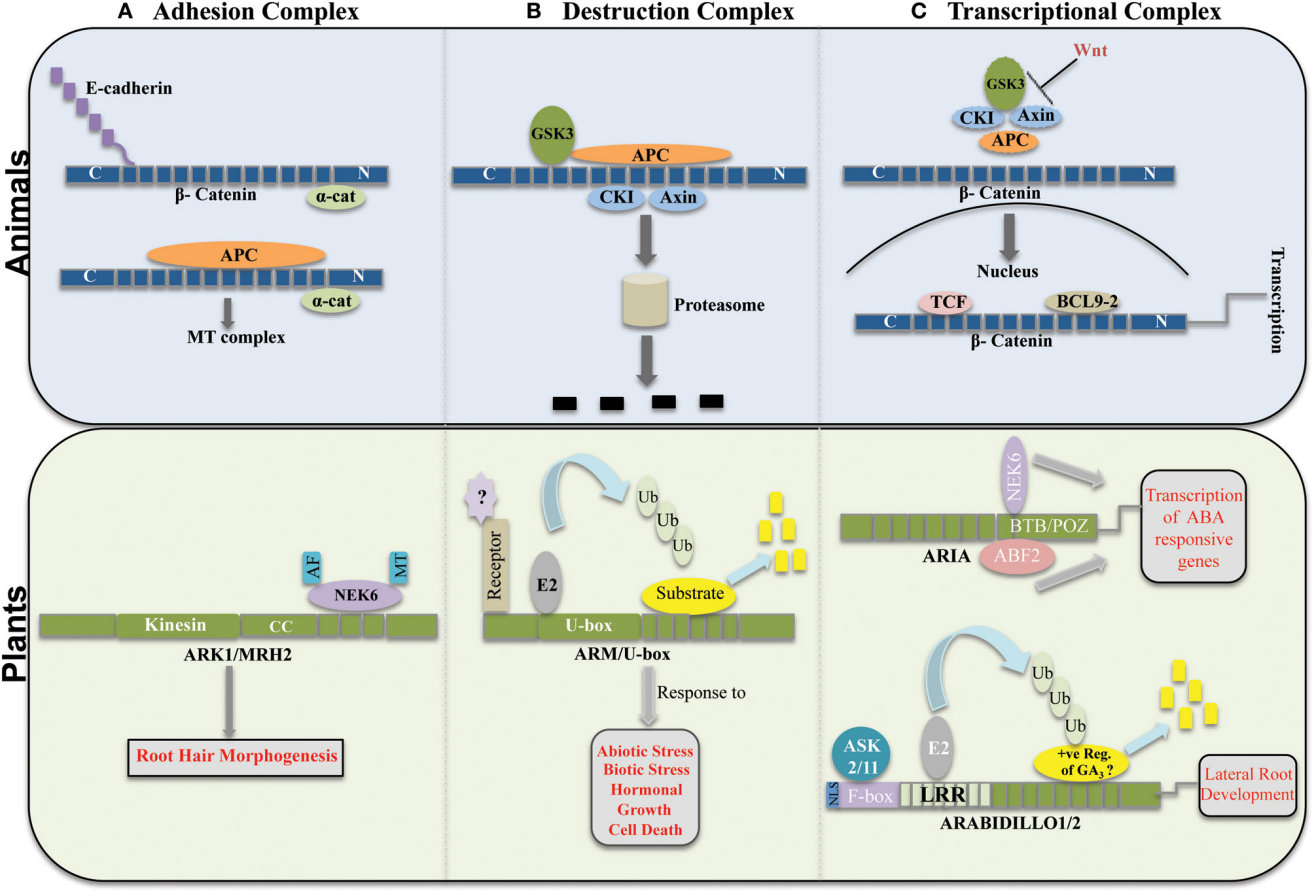
**Functional comparison of β-cat like-ARM repeats protein in plants and animals. (A)** Adhesion Complex: β-catenin in animals binds cytoplasmic tail of cadherin to link it with α-catenin. Additionally, β-catenin together with APC interacts with microtubule complexes. In plants, ARK1/MRH2 (ARM repeat kinesin1/morphogenesis of root hair 1) interacts with NEK6 (NIMA-related protein kinase 6) to mediate root epidermal cell morphogenesis. CC represent coiled coil domain. **(B)** Destruction Complex: β-catenin is targeted for proteasomal degradation by a GSK3, APC, CKI, and Axin complex in the cytoplasm. Similarly in plants, ARM/U-box proteins, in response to various stimuli target substrate protein for proteasomal degradation. **(C)** Transcriptional Complex: Wnt signals inhibits the destruction complex, free β-catenin enters the nucleus where it links with the transcriptional regulators to activate transcription of target genes. In plants, ARIA an ARM protein with BTB/POZ domain binds with ABF2 and NEK6 transcription factors to stimulate transcription of ABA responsive genes. Additionally, ARABIDILLO1/2 interacts with ASK2/11 through their F-box domain to mediate degradation of possibly a positive regulator of GA3 signaling to promote transcription of genes related to lateral root development.

Wnt proteins are one of the foremost signaling molecule essential for cell polarity, embryonic development and the determination of cell fate in metazoa (Cadigan and Nusse, 1997; Wodarz and Nusse, 1998; Logan and Nusse, 2004). A combination of molecular and genetic studies has provided evidences for how Wnt1, Wnt3a, and Wnt8 specifically induce the activation of “canonical β-catenin” pathway in animals (Du et al., 1995; Shimizu et al., 1997; Kuhl et al., 2000). However, no evidence for a Wnt, Frizzled (Fz) and low-density-lipoprotein-related protein receptors has been obtained in plants. Despite this, few homologs of proteins, which act as negative regulator of Wnt signaling has been unveiled in plants. Based on BLAST searches, the serine/threonine kinase GSK-3 (glycogen synthase kinase-3), CK1 (casein kinase 1) and APC (Adenomatous polyposis coli), which together form a destruction complex to stimulate degradation of β-catenin in animals were found to be conserved in plants (Figure 1) (Li et al., 2001). This has been proven in animals that activity of GSK3/CK1 complex is inhibited in response to Wnt signal perception at the cell surface to relieve its inhibitory effects on downstream β-catenin (He et al., 2004; Tamai et al., 2004; Nusse, 2005). The conservation of β-catenin destruction complex in plants points toward novel targets and modulation of Wnt signaling.

## Potential “Wnt-like” signaling functions for plant ARM family proteins

Arabidopsis comprises a multigene SHAGGY-related protein kinase (ASK) gene family, which is 70% identical to glycogen synthase kinase-3 from mammals, (Bourouis et al., 1990; Siegfried et al., 1990; Woodgett, 1990) classified into four distinct subfamilies (Jonak and Hirt, 2002). In the past few years, significant progress has been made in understanding how GSK3s perform their diverse functions in plants. The diverged biological functions of these members in signal transduction, cell patterning, cytokinesis and determination of cell fate has been established and credited to their diversity within plants (Dornelas et al., 1998). Most of the plants GSKs are found to be involved in brassinosteroid signaling and salt stress response (Dornelas et al., 2000; Kim et al., 2009). Brassinosteroids (BRs) are plant hormones, which signal through a plasma membrane localized receptor kinase BRI1. BRI1 interacts with BAK1 (BRI1 associated receptor kinase 1) to mediate plant steroid signaling (Nam and Li, 2002). BES1 has been identified as a suppressor of BRI1, which in turn is negatively regulated by a kinase BIN2 (Yin et al., 2002). Interestingly, the BR signaling pathway mechanism is analogous to the Wnt signaling pathway. In the proposed model, BIN2 which shares sequence homology with GSK-3 (Li and Nam, 2002), phosphorylate and destabilize its substrate BES-1. In response to brassinosteroids, BES-1 is stabilized and accumulates in the nucleus to activate target gene expression (Yin et al., 2002).

It is important to note that both BES-1 and β-catenin does not share homology at the protein sequence level. Similarly, BRI1 and Wnt are the two different receptors and does not belong to the same family (He et al., 2002; Yin et al., 2002; Zhao et al., 2002). However, it will be interesting to know if any of the protein in multigene Armadillo family in plants, gets regulated in the same manner or it is simply the way in which the pathway is conserved.

Meanwhile, several lines of evidence suggest the role of Wnt signaling proteins i.e., Armadillo repeats containing proteins in the developmental regulation in both animals and plants (Amador et al., 2001). p120ctn is an Armadillo repeat protein identified as a component of E-cadherin-catenin cell adhesion complex (Daniel et al., 2002). The signaling and cell adhesion co-factor p120ctn is the only known binding partner for Kaiso, a novel BTB/POZ domain zinc finger transcription factor (Daniel et al., 2002). Another possible candidate mediating interaction within actin and microtubule filaments in plants is ARK/MRH2 kinesin (ARM repeat kinesin/Morphogenesis of root hair). ARK/MRH2 interacts with NIMA-related protein kinase NEK6, to regulate epidermal cell morphogenesis by modulating microtubule dynamics (Sakai et al., 2008).

In relation to this, Arabidopsis (AT5G13060) and rice (LOC_Os05G33050) also possess homologous proteins comprising ARM repeats and a BTB/POZ domain (Figure 1). The Arabidopsis BTB/POZ ARM protein also known as ABAP1 has been shown to be involved in DNA replication and gene transcription controls (Masuda et al., 2008).

*Arabidillo-1/-2* and *Oryzadillo* are the closest homolog of β-catenin in Arabidopsis and *Oryza sativa* respectively, consisting of an F-box motif near their N-terminal, and several presumed sites for GSK-3 phosphorylation (Gagne et al., 2002; Kuroda et al., 2002; Coates, 2003). Remarkably, Arabidillo's are closest to the β-catenin homolog in Dictyostelium' Aar protein that consists of an F-box domain and is required for the differentiation and expression of prespore specific genes (Grimson et al., 2000). Besides, analogous to animals, physical interaction of Arabidillo-1/-2 proteins through their F-box domain with ASKs (SHAGGY-like protein kinase) lead to the formation of SCF complexes that target various substrates for ubiquitn/26S proteasome–mediated proteolysis has been proven in plants (Changjun et al., 2010). This suggest an evolutionary conservation of signal transduction pathway elements and their site of action within animals and plants.

## Beyond Wnt signaling: role of plant ARM proteins

Exposure to abiotic and biotic stress results in alteration of cellular homeostasis in plants. The first response to stress factors, is to activate the signal transduction pathways that stimulate cell defense and adaptive mechanisms. Ubiquitination is a unique protein degradation mechanism utilized by plants to effectively degrade detrimental cellular proteins and components specific to these stress signalings. A majority of U-box E3 ubiquitin ligase encoding ARM proteins related to biotic and abiotic stress have been identified in plants. We can certainly anticipate new insight into the molecular mechanism of plant β-catenin-like proteins function in the context of abiotic stress signals.

There are 41 and 47 predicted U-box/ARM proteins in the genome of Arabidopsis and rice respectively (Mudgil et al., 2004; Sharma et al., 2014). A few of them have been functionally characterized in Arabidopsis. Many of these proteins have now been linked to specific stress and hormonal responses.

A biological role for the U-box/ARM protein *AtPUB9* has been proposed in ABA (Abscisic acid) signaling (Samuel et al., 2008). In Arabidopsis, ATPUB18 and ATPUB19 are the two homologous proteins. Molecular analysis of *AtPUB19* showed that it is upregulated in response to drought, salt, cold and ABA (Liu et al., 2011). In the consecutive year, role of *ATPUB18* as a negative regulator has been put forward in ABA-mediated stomatal closure and drought responses (Seo et al., 2012). A different homologous pair of PUB proteins, AtPUB22 and 23 have been shown to play a combinatory role in the negative regulation of drought stress (Cho et al., 2008; Seo et al., 2012). A closely related ortholog of *ATPUB22/23* in *Capsicum annum* known as *CaPUB1* was found to be highly inducible in response to various abiotic stresses such as drought, cold and salt (Cho et al., 2006).

Another report suggested the role of AtCHIP, an Arabidopsis U-box/ARM protein in response to extreme temperature conditions. Subsequently, AtCHIP was reported to be involved in the ABA stress signaling pathway by mediating interaction with protein phosphatase 2A (Yan et al., 2003). In rice, SPL11 was identified as a U-box containg ARM protein that functions as a negative regulator in the control of cell death and pathogen defense (Zeng et al., 2004). The Arabidopsis ortholog of SPL11, ATPUB13 is a functionally conserved protein regulating plant defense, cell death and flowering time (Li et al., 2012a,b). In *Nicotiana*, two U-box/ARM proteins NtCMPG1 and tobacco ACRE276 and their functional homolog in Arabidopsis, AtPUB17 has been implicated as positive mediators of plant defense and stress signaling (Gonzalez-Lamothe et al., 2006; Yang et al., 2006). Apart from this, expression analysis in rice has confirmed many of the ARM proteins without any associated domain to be differentially regulated under abiotic stress conditions suggesting a role of ARM repeats in the stress regulation (Sharma et al., 2014).

On the basis of facts described above, it can be concluded that animal and plant ARM repeat proteins share many resemblances. Therefore, it is possible that at least some transcription effectors involved in Wnt signaling are evolutionary conserved. These elements include nuclear accumulation in response to extracellular signal, phosphorylation and degradation. Apart from the common response, plants possess specific signaling pathways mediated by ARM proteins. In plants, ubiquitination is critically involved in the function of ARM proteins. The proliferation of β-catenin-like ARM proteins in plants suggest their significance in the regulation of diverse biological fuctions in them. Further study of these proteins in plants would contribute to our understanding of the molecular factors involved in response to abiotic stress.

### Conflict of interest statement

The authors declare that the research was conducted in the absence of any commercial or financial relationships that could be construed as a potential conflict of interest.
